# Costs of missed work among employed people with inflammatory bowel disease: a cross-sectional population-representative study.

**DOI:** 10.23889/ijpds.v7i3.1918

**Published:** 2022-08-25

**Authors:** Ellen Kuenzig, Michael Lebenbaum, Joyce Mason, Manav Vyas, Gilaad Kaplan, Sanjay Murthy, Laura Targownik, Eric Benchimol

**Affiliations:** 1 SickKids Research Institute; 2 University of Toronto; 3 The Centre for Addiction and Mental Health; 4 University of Calgary; 5 University of Ottawa; 6 The Hospital for Sick Children

## Objectives

Inflammatory bowel disease (IBD) is a chronic inflammatory disease primarily affecting the gastrointestinal tract. Periods of relapsing and remitting abdominal pain and diarrhea result in decreased quality of life. We report the impact of IBD on missed work and associated costs using population-representative data to understand population-level indirect costs.

## Approach

We used the 2014 Canadian Community Health Survey to compare missed work and associated costs among employed respondents aged 20 to 64 years with and without self-reported physician-diagnosed IBD. Students and individuals with non-IBD bowel disorders were excluded. Costs of missed work were derived from reported annual income and the number of workdays missed. Survey weights were used for descriptive statistics. Logistic regression compared odds of employment. Negative binomial models compared the number of days missed and the cost of lost work in the two groups. All costs are in Canadian dollars and adjusted for inflation to 2022 dollars.

## Results

Among 33,344 eligible respondents, 1.0% had IBD. Employment in the last 3 months was reported among 72% of people with IBD and 81% of people without IBD (OR: 0.60, 95% CI 0.59-0.74). During this time, 48% of people with IBD and 31% of those without IBD missed work with a mean (sd) of 1.99 (5.17) and 1.03 (3.67) days of work missed, respectively (RR for days missed: 2.19, 95% CI 1.63-3.03). This corresponds to a cost of $608 (1635) among individuals with IBD and $298 (1063) among those without IBD every 3 months (RR: 2.18, 95% CI 1.22-4.43). Extrapolating to the estimated 133,030 employed Canadians with IBD, the attributable indirect cost of missed work due to IBD is nearly $165 million annually.

## Conclusion

The economic burden of IBD resulting from missed work is substantial. Since IBD is most often diagnosed during adolescence and early adulthood, employability and workplace productivity are important long-term outcomes for patients. Future interventions should be developed to help address these challenges.

